# Precision design of Ti sites for unprecedented catalytic performance

**DOI:** 10.1093/nsr/nwae172

**Published:** 2024-05-27

**Authors:** Haoquan Zheng, Shunai Che

**Affiliations:** Key Laboratory of Applied Surface and Colloid Chemistry, Ministry of Education, School of Chemistry and Chemical Engineering, Shaanxi Normal University, China; School of Chemistry and Chemical Engineering, Shanghai Jiao Tong University, China

In 1983, Taramasso *et al.* incorporated titanium (Ti) into porous silica frameworks to endow Ti sites with catalytic oxidation ability [1]. Nowadays, catalytic oxidation reactions using titanium silicate materials have become one of the most important processes in the petrochemical industry [2,3]. Among them, catalytic oxidative desulfurization (ODS) has emerged as a promising technique for removing thiophenic sulfurs from fuels to meet stringent environmental regulations [4]. Microporous titanium silicates such as titanium silicalite-1 (TS-1) with framework tetra-coordinated Ti (TiO_4_) sites are normally used as ODS catalysts. However, TS-1 catalysts have limitations due to their narrow pore structure (<0.56 nm), which severely restricts the diffusion and transport of bulky thiophenic sulfurs to access Ti active sites [5]. In recent years, framework hexa-coordinated Ti (TiO_6_) sites have been revealed to have superior activity in the oxidation of certain alkenes and thiophenic sulfurs compared with TiO_4_ sites [6,7]. Unfortunately, the precise control of uniform framework TiO_6_ sites in titanium silicates remains highly challenging.

In the paper entitled ‘Engineering surface TiO_6_ single sites for unprecedented catalytic ODS’ by Profs Li-Hua Chen and Bao-Lian Su, the authors achieve, for the first time, the precise location of highly accessible and active TiO_6_ single sites on the mesopore surface using an innovative method involving monitoring the electrostatic interface during the synthesis of mesoporous materials [8]. This groundbreaking research, exemplifying ‘materials by design’, brings three key breakthroughs.

For the first time, the authors conducted density functional theory investigations on the catalytic oxidation of bulky thiophenic sulfurs. They found that, compared with the TiO_4_ site, the TiO_6_ site can interact with oxidant ((CH_3_)_3_COOH) via a hydrogen bond network and orient a much lower-energy pathway of the ODS process due to the significantly lower adsorption energy of the oxidant (–78.7 kJ/mol) than that of the framework TiO_4_ sites (−22.2 kJ/mol).

**Figure 1.**
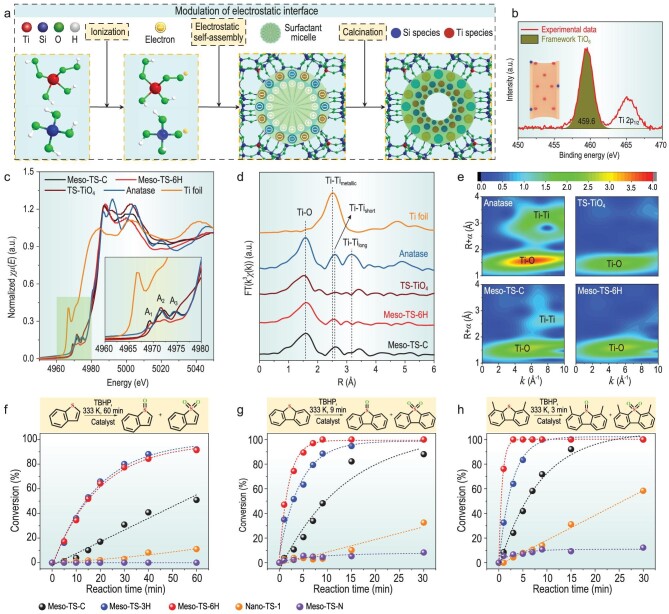
(a) Schematic of the proposed method of modulating the electrostatic interface during the synthesis of mesoporous titanium silicate material. (b) X-ray photoelectron spectroscopy spectrum of Ti 2p_3/2_, (c–e) X-ray absorption fine structure results and (f–h) catalytic oxidative desulfurization performance of the obtained mesoporous material. Adapted from Ref. [8].

Guided by the theoretical results, the researchers developed an innovative synthesis method to create highly accessible and uniformly distributed framework TiO_6_ single sites on the mesopore surface by engineering the electrostatic interface between negatively charged Ti species and positively charged surfactant molecules. As depicted in Fig. 1a, a unique TiOOH species possessing significantly enhanced ionization ability compared with the conventional hydrolysis product TiOH was used as a new hydrolysis product. This groundbreaking idea is based on the fact that the first ionization constant of H_2_O_2_ (*K*_1_ = 1.55 × 10^–12^) is 155 times higher than that of H_2_O (*K*_1_ = 1.00 × 10^–14^). Considerable negatively charged TiOO^−^ interacted with positively charged surfactants at the electrostatic interfaces during an electrostatic self-assembly process. After the completion of the electrostatic self-assembly and subsequent calcination treatment, the surfactants were removed to produce mesopores, while TiOO^−^ at the interface transformed into TiO_6_ sites on the mesopore surface. Only TiO_6_ sites were found on the surface of the obtained mesoporous materials (Fig. 1b). These Ti sites were further evidenced to be highly coordinated, asymmetrical single TiO_6_ sites (Fig. 1c–e). Systematical investigations were further conducted and confirmed the open and highly accessible space around these TiO_6_ sites.

The novel mesoporous titanium silicate materials developed in this work exhibited extraordinary ODS performance (Fig. 1f–h), achieving complete removal of benzothiophene (BT), dibenzothiophene (DBT) and 4,6-dimethyldibenzothiophene (DMDBT) from model fuels. Under a reaction at 60°C for 60 minutes, this catalyst reached a BT conversion of 920 ppm, which was 1.67 times that of the best catalyst reported so far. Even for a bulky molecule such as DMDBT, it took only 3 minutes to achieve a conversion of 500 ppm at 60°C, which was five times faster than that with the current best catalyst. These results strongly evidence that TiO_6_ sites are superior catalytic centers for ODS, consistently with the work reported by Yu's group [9]. This catalyst showed great reusability with highly stable activity upon five reaction cycles, attributed to the significantly improved structural stability resulting from an enhanced electrostatic self-assembly process.

This work illustrates a concrete example of ‘Materials by Design’ for sustainable industrial processes with highly enhanced efficiency. It is noteworthy that the developed synthesis method is cheap and easily scalable for the large-scale production of mesoporous titanium silicate catalysts for use in industrial deep ODS of bulky sulfur compounds.
